# The role of immune cell signatures in the pathogenesis of ovarian-related diseases: a causal inference based on Mendelian randomization: a correspondence

**DOI:** 10.1097/JS9.0000000000003145

**Published:** 2025-08-07

**Authors:** Jinling Yi, Seyida Yimamuyushan, Song Shi

**Affiliations:** Department of Gynecology, The Fifth Affiliated Hospital of Xinjiang Medical University, Urumqi, China

*Dear Editor,*

The paper is in compliant with the TITAN Guidelines 2025—governing declaration and use of AI^[1]^. AI (Tengxun Yaunbao- hunyuan) was used for checking the English writing style and finding grammatical errors, and no patient data was provided to AI during the preparation of this manuscript.

Lu et al.’s study represents a pioneering effort to dissect the genetic interplay between immune cell phenotypes and ovarian pathologies using Mendelian randomization (MR) and Bayesian colocalization (COLOC)^[2]^. While the authors acknowledge several limitations, including population stratification and reliance on cross-sectional data, additional critical gaps warrant attention to fully contextualize the findings and guide future research directions.

## Nonlinear dynamics and environmental interactions

The study’s MR framework assumes linear relationships between immune traits and disease outcomes, yet immune processes in ovarian diseases likely involve nonlinear interactions with metabolic, hormonal, or environmental factors^[3]^. For example, estradiol modulates CD4+ T-cell polarization, and chronic inflammation may synergize with genetic predispositions in PCOS pathogenesis. Future studies should incorporate multivariable MR or mediation analyses to dissect how immune-ovarian axes are shaped by contextual factors like diet, endocrine disruptors, or microbiome composition.

## Temporal dynamics of immune dysregulation

By leveraging cross-sectional GWAS data, the study cannot distinguish between primary immunologic dysregulation and compensatory responses secondary to disease progression. For instance, elevated plasma blast counts in POF might reflect both autoimmune activation and reparative mechanisms. Longitudinal immune profiling, ideally combined with single-cell transcriptomics, is needed to parse dynamic trajectories of immune cell subsets, such as regulatory T-cell exhaustion in POI, and their temporal alignment with ovarian dysfunction.

## Mechanistic gaps in colocalization findings

While COLOC identified shared genetic loci (*PLXNA2* in PCOS), the study stops short of linking these variants to functional consequences for immune cell behavior or ovarian tissue. For example, rs575687159’s proximity to *PLXNA2*, a gene implicated in axon guidance^[4]^, raises questions about neuroimmune communication in ovarian homeostasis. Experimental validation using CRISPR-Cas9 knockout or in vitro co-culture systems is essential to bridge the gap between statistical colocalization and biological mechanism.

## Ovarian microenvironment heterogeneity

The analysis focuses exclusively on circulating immune cells, overlooking tissue-resident immune populations critical for ovarian homeostasis. Single-cell studies reveal distinct macrophage subsets in the ovarian follicular niche, which exhibit anti-inflammatory phenotypes distinct from their systemic counterparts^[5]^. Integrating spatial transcriptomics or imaging mass cytometry could elucidate how local immune-epithelial crosstalk contributes to diseases like ovarian cysts or neoplasms.

## Translational relevance and clinical validation

Though the study identifies 28 immune biomarkers, the absence of validation in independent cohorts or functional assays limits clinical utility. For example, CD4 + CD8dim T cells’ protective role in benign ovarian tumors requires replication in prospective cohorts and mechanistic studies such as adoptive transfer in murine models. Moreover, the study’s focus on European ancestry restricts generalizability; multiethnic validation is critical given HLA allele frequency disparities across populations^[6]^.

In sum, Lu *et al*’s work lays a foundation for immunogenetic research in ovarian diseases, but addressing these gaps is essential for translating genetic insights into precision medicine. Future studies should adopt longitudinal, multi-omics approaches and integrate functional validation to unravel the complex choreography between immune cells and ovarian tissues.

## Data Availability

There is no data involved in the manuscript.

## Research registration unique identifying number (UIN)

This is not registered since this is a commentary about a published article.
